# Signature Evaluation Tool (SET): a Java-based tool to evaluate and visualize the sample discrimination abilities of gene expression signatures

**DOI:** 10.1186/1471-2105-9-58

**Published:** 2008-01-28

**Authors:** Chih-Hung Jen, Tsun-Po Yang, Chien-Yi Tung, Shu-Han Su, Chi-Hung Lin, Ming-Ta Hsu, Hsei-Wei Wang

**Affiliations:** 1 Microarray & Gene Expression Analysis Core Facility, VGH National Yang-Ming University Genome Research Center, Taipei, Taiwan; 2 Institute of Microbiology and Immunology, National Yang-Ming University, Taipei, Taiwan; 3 Institute of Biochemistry and Molecular Biology, National Yang-Ming University, Taipei, Taiwan; 4 Department of Teaching and Research, Taipei City Hospital, Taipei, Taiwan; 5 EMBL-European Bioinformatics Institute, Wellcome Trust Genome Campus, Hinxton, Cambridge CB10 1SD, UK

## Abstract

**Background:**

The identification of specific gene expression signature for distinguishing sample groups is a dominant field in cancer research. Although a number of tools have been developed to identify optimal gene expression signatures, the number of signature genes obtained is often overly large to be applied clinically. Furthermore, experimental verification is sometimes limited by the availability of wet-lab materials such as antibodies and reagents. A tool to evaluate the discrimination power of candidate genes is therefore in high demand by clinical researchers.

**Results:**

Signature Evaluation Tool (SET) is a Java-based tool adopting the Golub's weighted voting algorithm as well as incorporating the visual presentation of prediction strength for each array sample. SET provides a flexible and easy-to-follow platform to evaluate the discrimination power of a gene signature. Here, we demonstrated the application of SET for several purposes: (1) for signatures consisting of a large number of genes, SET offers the ability to rapidly narrow down the number of genes; (2) for a given signature (from third party analyses or user-defined), SET can re-evaluate and re-adjust its discrimination power by selecting/de-selecting genes repeatedly; (3) for multiple microarray datasets, SET can evaluate the classification capability of a signature among datasets; and (4) by providing a module to visualize the prediction strength for each sample, SET allows users to re-evaluate the discrimination power on mis-grouped or less-certain samples. Information obtained from the above applications could be useful in prognostic analyses or clinical management decisions.

**Conclusion:**

Here we present SET to evaluate and visualize the sample-discrimination ability of a given gene expression signature. This tool provides a filtration function for signature identification and lies between clinical analyses and class prediction (or feature selection) tools. The simplicity, flexibility and brevity of SET could make it an invaluable tool for marker identification in clinical research.

## Background

Gene expression profiling based on microarray technology has been applied widely on monitoring global transcriptome changes in biological samples. In cancer research, one of the major microarray applications is to identify genes, or features, whose expression patterns can discriminate samples with distinct states (usually defined by the phenotype of samples such as primary or metastatic tumour). These identified genes form an expression signature that can be used to assist clinical management decisions such as clinical trail risk assessment, treatment selection, or cancer prognosis [1-5].

To acquire a good expression signature, supervised methods are more appropriate than unsupervised approaches. Basically, a supervised prediction method consists of three common processes: 1) feature selection, 2) computation of weights for selected features, 3) creation of a prediction rule [6]. By using the cross-validation method such as n-fold or leave-one-out cross-validation (LOOCV), the discrimination capability of a signature can be evaluated. Recently, many classification algorithms (such as SVM, evolutionary algorithm and I-RELIEF) combining cross-validation and heuristic searching to acquire an optimal expression signature have been proposed [7-9]. Furthermore, those algorithms have been incorporated into hassle-free tools to aid the acquisition of an optimal signature. For example, M@CBETH [10] is a web-based tool aimed at finding the best prediction among different classification methods. Prophet [11], another web-based tool, can automatically build classifiers using a strategy that renders unbiased cross-validated errors. The class prediction modules in GenePattern [12] also supports several supervised learning methods. Moreover, for improving the efficiency and the accuracy of an acquired signature, several feature selection tools based on statistical analysis have been developed: RankGene is a feature selection suite based on statistical ranking analyses [13], HykGene [14] and mRMR [15] are tools to minimise redundancy of genes.

**Figure 1:**
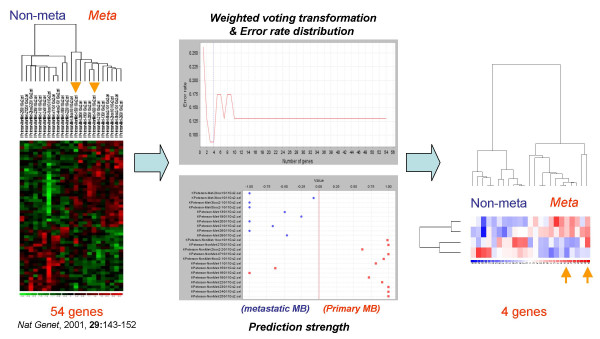
**Narrowing down existing gene signature to few genes**. 23 human medulloblastoma expression profiles implemented by Affymetrix G110 cancer arrays were used [16]. Among them, 10 were metastatic tumours and 13 were non-metastatic tumours. This plot illustrates how users can filtrate out a handful of genes for diagnostic purpose by applying SET. The left panel is the heat map of the original signature [16], and arrows indicate mis-grouped samples. The middle panel shows two figures produced by SET (see **Figure 2 **for more details). The right panel is the heat map of the filtrated 4 new diagnostic markers.

Although the aforementioned feature selection and classification tools are quite useful for acquiring an optimal signature, a tool assisting signature evaluation is still in high demand. In clinical practice, the ability to distinguish a patient group from others based on a smaller number of specific genes is of tremendous value and, thus, tools that assist to narrow down on candidate genes (see Figure 1 as an example) is central to the identification of unique signatures. On the other hand, it is sometimes desired to investigate the discriminative power of genes of interest, such as those deduced from biological experiments or, perhaps, based on other consideration such as the availability of antibodies and reagents. Bearing these in mind, we developed a simple and flexible Java standalone tool, the Signature Evaluation Tool (SET), to fulfill the needs of clinical evaluations. SET both accepts and creates a "user-defined" signature and then utilize a visualization module to present the classification consequences. SET not only accelerates the feature evaluation process but can also predict the groups of unknown samples.

In SET, we adopted the weighted voting algorithm published by Ramaswamy *et al*. and LOOCV [3,16,17] to evaluate the discrimination power of features. The signal-to-noise score was used:

**S**_**x**_**= (μ**_**GI **_**- μ**_**GII**_**)/(σ**_**GI **_**+ σ**_**GII**_**)**

**S**_**x**_: the weighted value for the each feature **x**

**μ**: mean of expression in group I (_**GI**_) or group II (_**GII**_)

**σ**: standard deviation of expression in group I (_**GI**_) or group II (_**GII**_)

The signal-to-noise statistics reflects how well a feature correlates with a particular group distinction (numerator). Also, it penalises features which have higher variance in both groups more than those having high variance in one group but low variance in another (denominator). This bias is useful for biological samples: for example, in cancer research, genes in normal tissues work normally and the regulation of which are strict. However, in tumours, genes are dysregulated and the levels of gene expression vary widely [18]. The weighted voting algorithm has been compared with other class prediction methods (standard and diagonal discriminant analysis, classification trees with or without aggregation, and nearest neighbour classification) using three microarray datasets (adult lymphoma, leukaemia, and sixty human tumour cell lines), and it was the one with the best performance [19].

In order to avoid over-interpretation of the error rate value produced by weighted voting algorithm, there is a visualization module in SET to present the prediction strength (PS) information for all samples [3,16,17,20]:

**PS = (V**_**GI **_**- V**_**GII**_**)/(V**_**GI **_**+ V**_**GII**_**)**

   **V**_**GI **_and **V**_**GII **_represent the total votes for **G**_**I **_and **G**_**II **_respectively

The PS value ranges from -1 to +1, with higher absolute values reflecting stronger prediction. The prediction strength for each sample shows the margin of victory in either direction of two supervised groups. The visualization of uncertainty will provide important information about prognosis, such as the progression of tumour metastasis or the estimated survival time [3].

## Implementation

SET is a standalone Java application that deploys Java Web Start technology, providing a flexible platform for researchers to evaluate gene signatures based on expression datasets. It enables users to analyze unpublished profiles locally with the most up-to-date version of the program. Results are visualized by JFreeChart, an open-sourced Java chart library, which displays the line chart of error rate distribution and the scatter plot of prediction strength analysis. This software exhibits several unique presentations and user-friendly elements by following four simple steps:

### Step1: Grouping arrays by supervised knowledge

**Figure 2:**
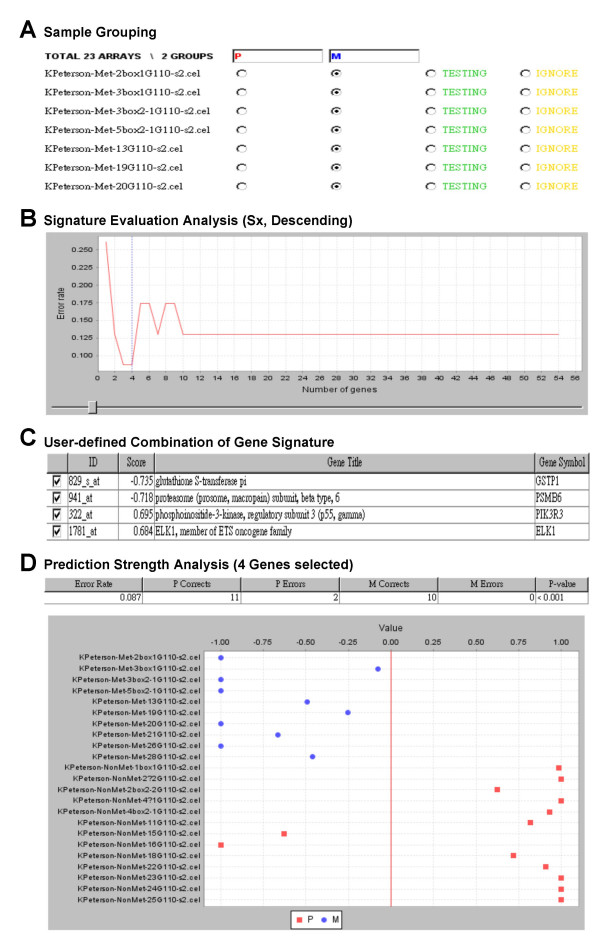
**Implementation of SET**. (**A**) 10 metastatic tumours and 13 non-metastatic tumours were assigned into M and P group, respectively. (**B**) Error rate distribution of expression signature genes. This plot suggested the top 3~4 genes are capable of being the best signature to distinguish samples (error rate 0.087). (**C**) "User-defining" interface allows user to select/de-select features. (**D**) The plot shows the prediction strength of a user-defined signature (top 4 genes from 2B) in discriminating non-metastatic and metastatic tumours. The table above PS plot shows the significance of the error rate (less than 0.001 in this case). (A) – (D) show the relevant sections of the original software interface. For the full images please see Additional files 1-4, respectively.

First, the user prepares and uploads two tab-delimited text files, one containing a gene expression matrix that has been normalised, filtered or transformed; and another containing a list of genes that are potential classification markers. In both files, individual genes (or probe IDs) are represented in rows while array samples or user-defined attributes are displayed in columns. To increase flexibility SET implements parsers to recognize a variety of popular data formats including normalised outputs from Expression Console™, BioConductor or dChip; and accepts published analytical results as gene list input or it can be user-defined. Upon uploading the files, array samples are assigned into two groups ("Supervised" groups) under the "Sample Grouping" panel. Samples of unknown identity can be assigned to the "Testing" group and their identities can be predicted in the latter step of prediction strength analysis. Samples to be excluded in latter analyses can be assigned to the "Ignore" group (Figure 2A).

### Step 2: Error rate distribution

By default, the uploaded genes are ranked according to the absolute values of corresponding signal-to-noise scores in a descending order, but can be user-defined to be ranked by other attributes such as p-values. Genes are included into a signature one at a time based on the order of ranking. The error rate for each new signature is estimated by the weighted voting algorithm and LOOCV [3] and can be monitored by an error rate distribution plot (Figure 2B). Subsequently, based on the error rate information, the user can select an appropriate composition of discriminating genes, for instance, a composition with the lowest error rate.

### Step 3: Signature evaluation

Genes within the chosen composition are ranked and displayed by their signal-to-noise scores and the user can manually select or de-select genes as appropriate (Figure 2C). Gene titles and gene symbols can be incorporated in this step if the annotations of an array platform are supported by our ArrayFusion database, which currently supports annotations for the majority of Affymetrix arrays and several Agilent arrays [21]. The potential of selected genes to distinguish between two supervised groups can be evaluated by cross-validating error rate information, where a lower error rate reflects a superior distinguishing potential. The significance of error rate is estimated by 1,000 times of group permutations to ensure that the error rate is not a result of random chance [22]. The expression signature can be arbitrarily modified during the analysis and the corresponding error rate can be recalculated repeatedly.

### Step 4: Prediction strength

The result of prediction strength (PS) analysis for each sample is shown once a signature is defined. The PS values range from -1 to +1, where higher absolute values reflect stronger predictions [17]. An overview of the results for samples in both "Supervised" and "Testing" groups is illustrated by the PS plot for the selected signature, and the results can be used to evaluate and predict the certainty of group identity for individual sample (see Figure 2D as an example). To increase the flexibility of evaluation, samples can be re-grouped (for instance, re-allocated from the "Testing" group to the "Supervised" group) and signature genes can be re-selected repeatedly (Figures 2A and 2C). Results of the analysis provide the user candidate genes for further experimental validation.

Further details are illustrated in the tutorial file on the website, please see the Availability and requirements section.

## Results and Discussion

### Serial signature evaluation

SET provides a rational way of narrowing down genes with optimal discriminative power. Unlike other feature selection tools such as Hykgene [14] or mRMR [15], which select non-redundant genes based on statistical calculations, SET adopts a speedy signature evaluation approach that ranks the gene list according to the contribution value (Sx or user-defined attributes) of individual genes and, additionally, plots the distribution of cross-validated error rate for signatures with increasing number of genes. Based on the distribution, the user can easily narrow down the number of genes with superior discriminative power; however, the approach is not without limitations. It is also possible that a subset of genes could generate lower error rate, albeit bearing lower weights. To avoid missing crucial genes, the user may commence by narrowing down the genes to a manageable quantity and subsequently select/de-select genes to further examine the power of the individual signature. Here, users are reminded that SET is a tool for signature evaluation rather than a machine learning tool for building an optimized prediction rule; in other words, the estimated error rate is only applied to the defined signature rather than to the signature building procedure that includes the feature selection process [6].

### A flexible evaluation platform

As described in Implementation (i) to (iii), SET provides a signature evaluation platform that can adapt signatures from a variety of sources including third party analyses or candidates of interest that are deduced by biological knowledge. The ability to re-select/de-select genes following error rate distribution analysis enables the user to further choose genes from the narrowed down list (Figure 2C), and rapidly re-evaluate and re-adjust the discriminative power of the new signature (Figures 2C and 2D).

With the accumulation of microarray experiments, researchers nowadays may have more than one gene expression dataset. To evaluate the applicability of a specific signature between different datasets, researchers can import two datasets into SET separately but select the same signature members to carry out the evaluation step. Alternatively, researchers can merge two datasets into one expression matrix, upload it into SET, and then perform a two-step evaluation procedure proposed by Gloub *et al*. [20]. In this case, one dataset can be set up as "Supervised" groups while the other as "Testing" group. The first step evaluation tests the selected features by cross-validation on samples of the "Supervised" group, and the second-step applies the built signature to assess its accuracy on the "Testing" group. Both results can be shown in the PS analysis.

**Figure 3:**
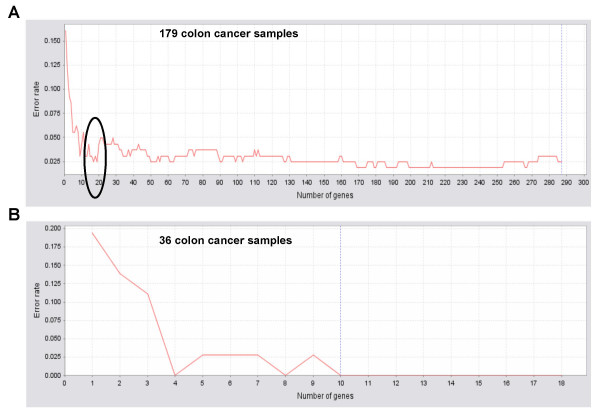
**Applying SET on two colon cancer datasets**. (**A**) Error rate distribution of 287 metastasis signature genes for 179 colon cancer arrays. The top 18 genes (indicated by a circle) had same discrimination power to that of over 130 genes. (**B**) Top 18 genes can also distinguish primary from metastatic colon cancer samples in another dataset (36 microarray samples) with p-value < 0.001.

Here, we demonstrate an example of applying SET to quickly identify diagnostic markers associated with colorectal carcinoma (CRC) metastasis. 179 Affymetrix U133 Plus 2.0 microarray data downloaded from the expO (Expression Project for Ontology) project (GSE2019; released before December 2006) were subjected to molecular signature analysis according to a pipeline we have previously used [23]. 287 genes were significantly (false discovery rate (FDR) q < 0.01) differentially expressed between primary and metastatic CRC. By further applying SET, it was found that the top 18 genes had a similar discriminative power to that of more than 130 genes (error rate 0.025; p-value < 0.001) (Figure 3A, indicated by a circle). Among those top 18 genes several were known metastasis markers, such as osteopontin and nexin [24,25], supporting the reliability of our result. When those 18 genes were applied to another dataset from expO (36 samples from GSE2109; release March 2007) they, again, had a good discriminative power (error rate 0; p-value < 0.001) (Figure 3B). By application of the two-step evaluation procedure on these two datasets, the error rates were 0.025 and 0.083, respectively (data not shown).

**Figure 4:**
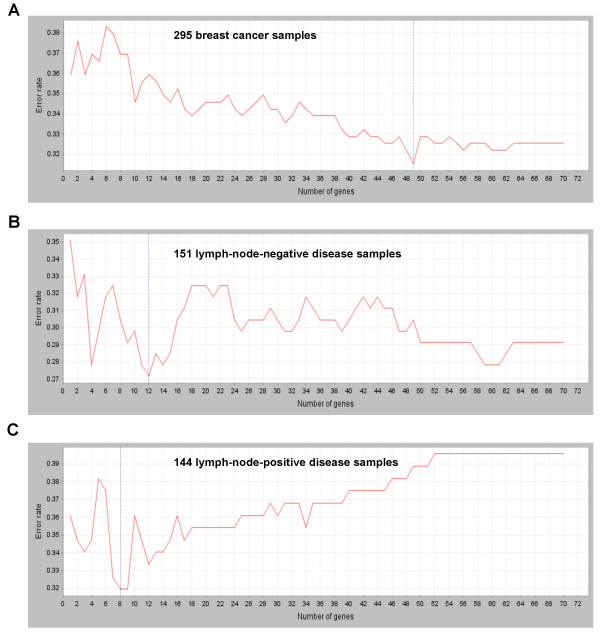
**Error rate distribution analysis for signatures related to breast cancer metastasis**. (**A**) Using 295 breast cancer samples (published by van de Vijver *et al*.), SET can reduce the gene number from 70 to 49 without losing prediction power (error rate 0.325 and 0.315, respectively). (**B**) For 151 samples with lymph-node-negative pathology status, the top 12 genes had a similar discrimination power to that of 70 genes (error rate 0.272 and 0.291, respectively; p value < 0.001). (**C**) For 144 samples with lymph-node-positive pathology status, the top 8 genes had a similar discrimination power to that of 70 genes (error rate 0.319 and 0.396, respectively; p value < 0.001).

To further validate the reliability of the tool, we further applied SET to analyse a signature of 70 genes related to breast cancer metastasis based on the data published by van de Vijver *et al*. [26]. Using the same 295 breast cancer samples SET reduced the gene number to a 49-gene signature without declination of prediction power (error rate 0.325 and 0.315, respectively; Figure 4A). We further divided the dataset into two smaller datasets according to their lymph node status from pathology report: among the 295 patients, 151 had lymph-node-negative disease (results of pathological examination) and 144 had lymph-node-positive disease [26]. For lymph-node-negative samples, we found the top 12 genes had a similar discriminative power to that of the 70 genes (error rate 0.272 and 0.291. respectively; p-value < 0.001). For lymph-node-positive samples, we found the top 8 genes had a similar discriminative power to that of 70 genes (error rate 0.319 and 0.396, respectively; p-value < 0.001). These results further consolidate the power of SET.

### Visualization of prediction strength (PS) for evaluation and prediction

Given that the prediction uncertainty is not revealed by estimation of error rate, the incorporation of the PS index in the analysis is of importance. The PS presentation methods used by MacDonald *et al*. [3,16,17] and Ye *et al*. [17] were integrated into SET, but a new way of displaying the PS information for each sample was devised (Figure 2D). The PS visualization module conveniently enables the user to trace back samples incorrectly grouped, or samples that have lower prediction certainty (PS value close to 0). Furthermore, the module would be of substantial value in clinical research when clinical parameters, such as disease progression, are taken into consideration. For example, in Figure 1, some primary tumour samples were grouped together with metastatic tumours. Not only does the tool enable re-validating the reliability of the features used, but also back tracking to the clinical information of those primary tumours, allowing potential discovery of patients with inferior clinical outcome or higher metastatic risk.

As described in Implementation, arrays in the same matrix can be annotated as "Supervised" or "Testing" samples in SET. Visualization of their PS information in the same plot enables the user to re-evaluate the discriminative power and validate the prediction power of a signature simultaneously.

### Application on multi-class datasets

For datasets containing multiple phenotypes, one-versus-all comparisons can be performed to filter associated markers. This strategy has been proven successful in several high-quality microarray experiments [27], and the incorporation of algorithms designed for multivariate issues into the next version of SET is currently in progress.

### SET and biological relevance analysis

Albeit it is of logic to assume biological correlation of signature genes between one another (for instance, the involvement in common pathways or genetic networks) the identification of the biological relevance of input or output genes, however, is not the primary function of SET. This tool is principally aimed at providing a gene filtration threshold for gene identification. Upon identification of a gene set of interest, the candidate genes can be applied to other biologically/clinically relevant analyses (such as Gene Ontology or Gene Set Enrichment Analysis) to determine the biological significance of those genes.

## Conclusion

SET provides a gene filtration threshold for gene identification between biological/clinical analyses and typical feature selection tools. SET is focused on the "evaluation" of input/selected genes to suggest their prediction/classification power. It rapidly narrows down candidate diagnostic markers from numerous signature genes and offer prediction information. The application of SET to filter out a smaller number of diagnostic markers from publically accessible databases was exemplified in this report. Taken together, the flexibility and reliability makes SET a valuable tool for various evaluations in clinical research.

## Availability and requirements

**Project name: **SET

**Project home page: **

**Operating system(s): **Platform independent

**Programming language: **Java and Java Web Start

**Other requirements: **Java 1.5.1 or higher

**License: **free

## Authors' contributions

HW conceived the tool is valuable for clinical cancer research. CJ, TY, CT, CL, MH, and HW suggested desired features and algorithmic approaches. CJ and TY carried out the implementation. CT, SS, and HW collected and analyzed microarray datasets. The online documentation and manuscript were written by CJ, TY and HW, and all authors read and approved the final manuscript.
